# Supplementary material for: The catalytic subunit of DNA polymerase δ inhibits γTuRC activity and regulates Golgi-derived microtubules
Source: Nat Commun. 2017 Sep 15;8:554. doi: 10.1038/s41467-017-00694-2 (PMC5601897; doi:10.1038/s41467-017-00694-2)
Supplement: Supplementary file 1 — Supplementary Information [file 41467_2017_694_MOESM1_ESM.pdf]

Description of Supplementary Files

Title: Supplementary Information

Description: Supplementary Figures and Supplementary Tables

Title: Peer Review File

Title: Supplementary Movie 1

Description: Golgi reorientation in control cells. Venus-β-1,4-galactosyltransferase (GalTase; green) was transiently expressed in RPE1 cells transfected with a control siRNA. For the recording, cells expressing the Golgi marker were selected from the first row of cells facing the wound edge. The play-back rate is 5 frames/s. Time is shown in the h:min format. Scale bar, 10 μm.

Title: Supplementary Movie 2

Description: Golgi reorientation in PolD1-depleted cells. Venus-GalTase (green) was transiently expressed in RPE1 cells transfected with a PolD1 siRNA. For the recording, cells expressing the green Golgi marker were selected from the first row of cells facing the wound edge. The play-back rate is 5 frames/s. Time is shown in the h:min format. Scale bar, 10 μm.

Title: Supplementary Movie 3

Description: Directional migration of control cells. A wound-healing assay was performed using RPE1 cells transfected with a control siRNA. After wounding, the cells were recorded once every 5 min for 20 h. The play-back rate is 15 frames/s. Time is shown in the h:min format. Scale bar, 20 μm.

Title: Supplementary Movie 4

Description: Directional migration of PolD1-depleted cells. A woundhealing assay was performed using RPE1 cells transfected with a PolD1 siRNA. After wounding, the cells were recorded once every 5 min for 20 h. The play-back rate is 15 frames/s. Time is shown in the h:min format. Scale bar, 20 μm.

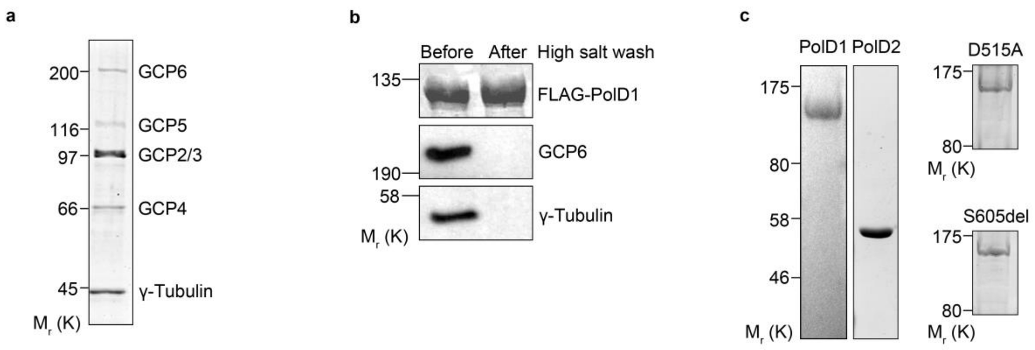

Supplementary Figure 1 Characterization of purified γTuRCs and FLAG-PolD1 proteins. (**a**) The SDS-PAGE gel image of purified γTuRCs was acquired on an imager (Typhoon Trio, GE Healthcare). The gel was stained with SYPRO Ruby. (**b**) FLAG-PolD1 was expressed in HEK293T cells and immunoprecipitated through the FLAG tag. Beads bound with the PolD1 protein were immunoblotted for γ-tubulin and GCP6 before and after washing under a stringent condition (1 M NaCl and 0.1% IGEPAL CA-630). After washing, the γ-tubulin complex proteins were not detectable on the beads. (**c**) FLAG-PolD1, its D515V and S605del mutants, and PolD2 were expressed and isolated as in (**b**). The PolD1 and PolD2 proteins were retrieved from the beads and then analyzed using SDS-PAGE and Coomassie Blue staining.

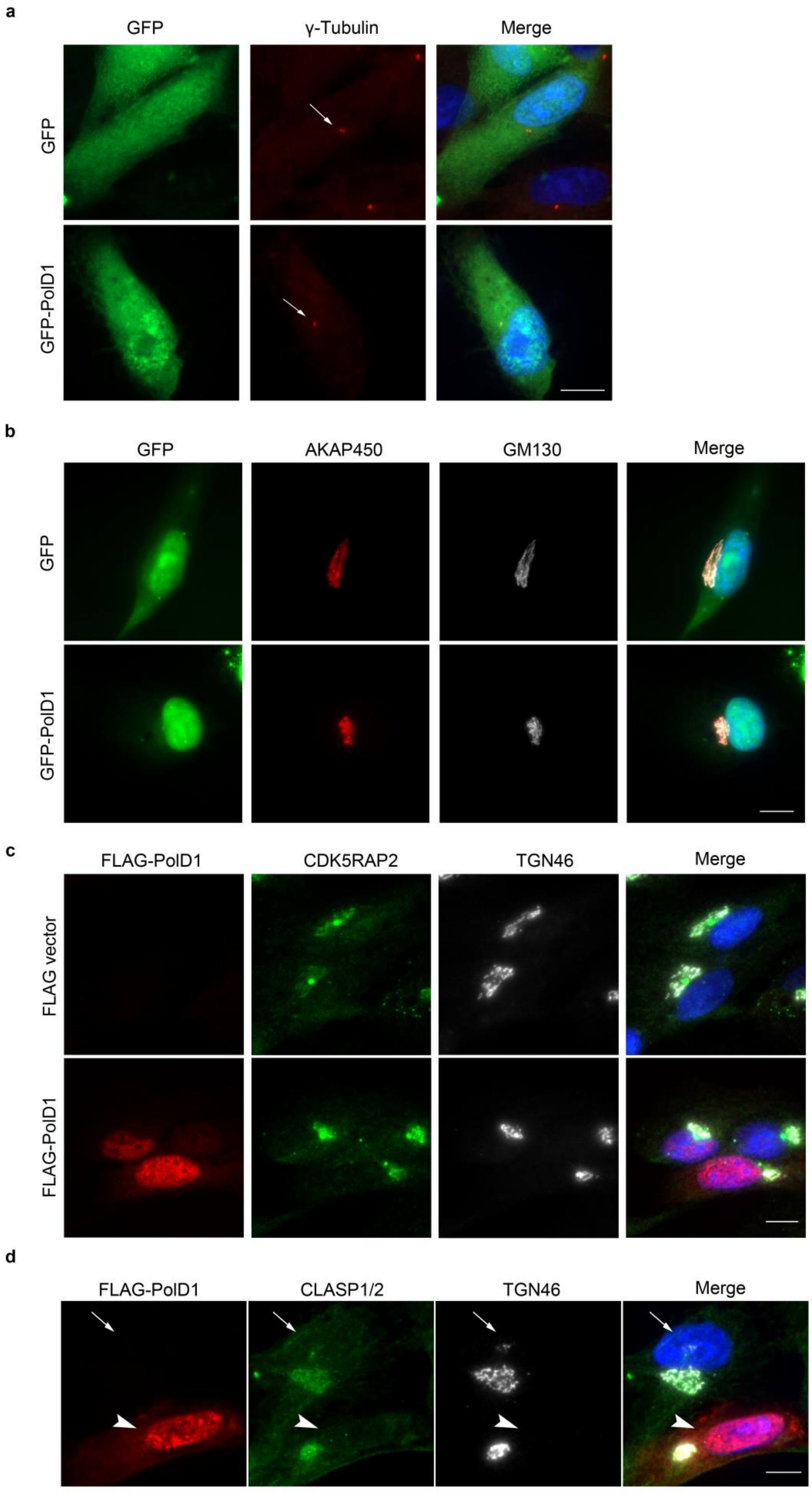

a
GFP
γ-Tubulin
Merge
GFP
GFP-PolD1
b
GFP
AKAP450
GM130
Merge
GFP
GFP-PolD1
c
FLAG-PolD1
CDK5RAP2
TGN46
Merge
FLAG vector
FLAG-PolD1
d
FLAG-PolD1
CLASP1/2
TGN46
Merge

Supplementary Figure 2 PolD1 overexpression does not affect the centrosomal level of γ-tubulin and the Golgi localization of AKAP450, CDK5RAP2, and CLASPs. RPE1 cells were transfected with PolD1-expressing constructs or their vectors, as indicated. The cells were immunostained for γ-tubulin (**a**), AKAP450 and GM130 (**b**), CDK5RAP2 and TGN46 (**c**), CLASP1/2 and TGN46 (**d**), as indicated. The nuclear DNA was stained with Hoechst 33258. (**a**) Arrows indicate γ-tubulin in transfected cells. (**d**) Arrows indicate a non-transfected cell, and arrowheads indicate a PolD1-transfected cell. The images shown are representative of at least 3 experiments. Scale bars, 10 μm.

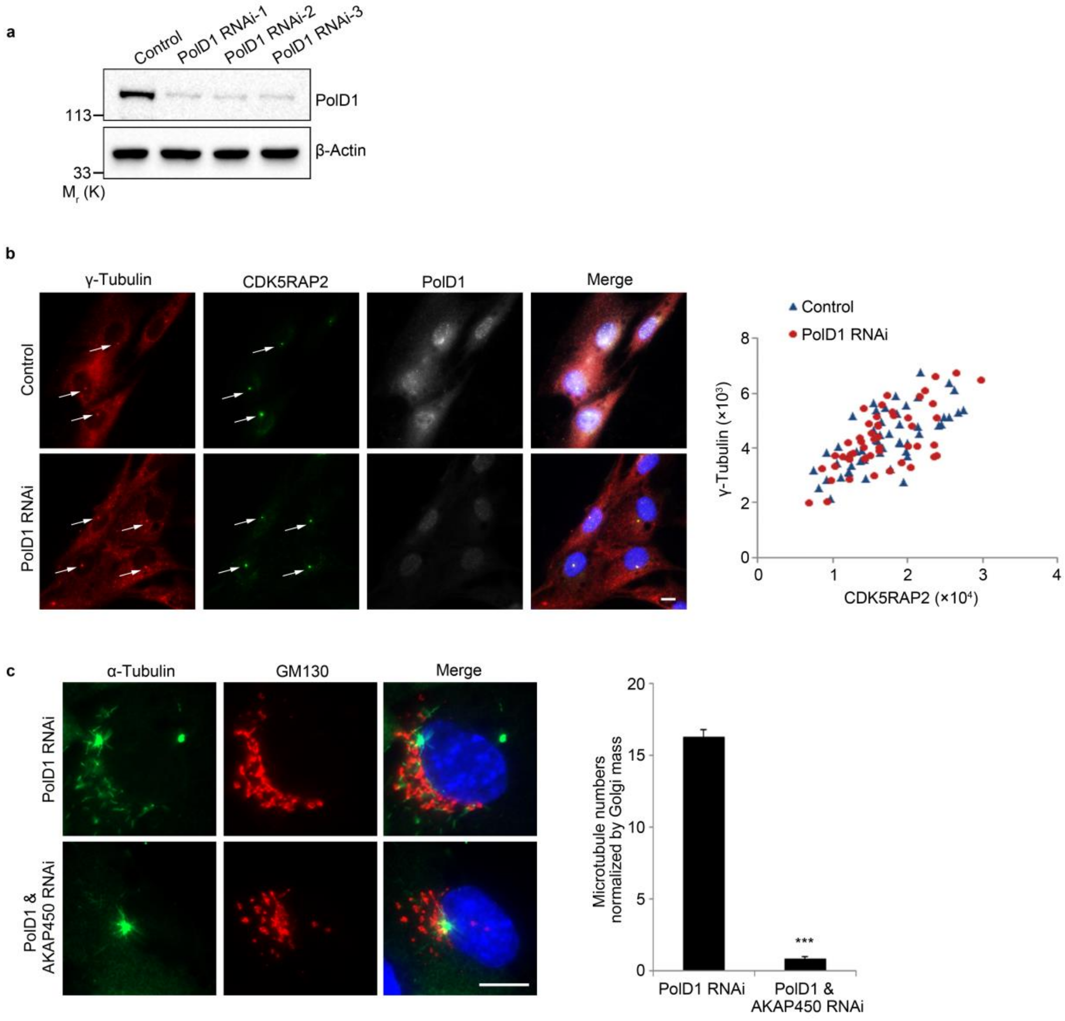

Supplementary Figure 3 Suppression of PolD1 expression through transfection of *pold1*-targeting siRNAs. (**a**) RPE1 cells were transfected with either *pold1*-targeting siRNA (PolD1 RNAi-1, PolD1 RNAi-2, or PolD1 RNAi-3) or control siRNA (Control), and 24 h later, the cells were analyzed by means of immunoblotting. Transfection of cells with each of the PolD1 siRNAs suppressed PolD1 expression by ~90%. (**b**) The centrosomal localization of γ-tubulin does not require PolD1. RPE1 cells were transfected with PolD1 siRNA (PolD1 RNAi) or control siRNA. The cells were fixed and immunostained for γ-tubulin and the centrosomal scaffold protein CDK5RAP2. Arrows indicate centrosomal γ-tubulin and CDK5RAP2. Hoechst 33258 was used to stain DNA. The images shown are representative of at least 3 experiments. The fluorescence intensities of γ-tubulin and CDK5RAP2 were measured within a circle (2 μm diameter)

centered at the centrosomes. The cytoplasmic fluorescence obtained using a circle of the same size was taken as the background and subtracted from the centrosomal fluorescence. (**c**) Suppression of AKAP450 expression inhibits PolD1-depletion-induced nucleation of Golgi-associated microtubules. After microtubule depolymerization at 0°C, cells were shifted to 37°C to allow microtubule regrowth for 1 min. Hoechst 33258 was used to stain DNA. After regrowth, Golgi-associated microtubules were counted and normalized by the Golgi mass. The images shown are representative of at least 3 experiments. Scale bars, 10 μm. The data are presented as means ± s.d. and are representative of 3 independent experiments; ***$p < 0.001$, two-tailed, unpaired student's t-test.

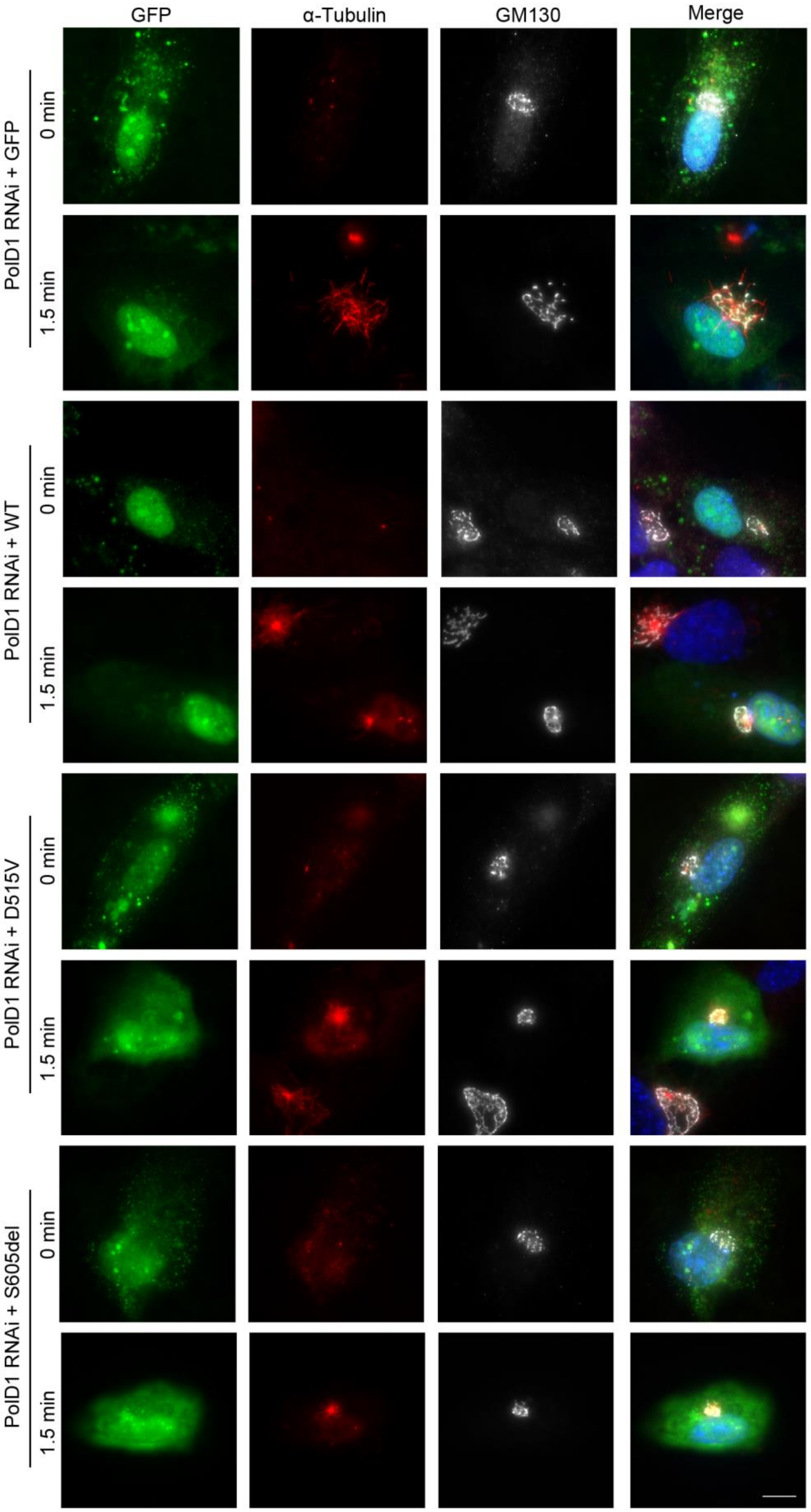

GFP
α-Tubulin
GM130
Merge
PolD1 RNAi + GFP
0 min
1.5 min
PolD1 RNAi + WT
0 min
1.5 min
PolD1 RNAi + D515V
0 min
1.5 min
PolD1 RNAi + S605del
0 min
1.5 min

Supplementary Figure 4 Microtubule regrowth induced by PolD1 depletion is inhibited by the expression of PolD1 or its D515V and S605del mutants. GFP-PolD1 (WT), the PolD1 mutants (D515V and S605del), and GFP were separately expressed in RPE1 cells that were transfected with the siRNA targeting an untranslated region of *pold1* (PolD1 RNAi-3). Microtubule regrowth was performed and then the cells were stained for microtubules (α-tubulin) and GM130. The images shown are representative of at least 3 experiments. Scale bar, 10 μm.

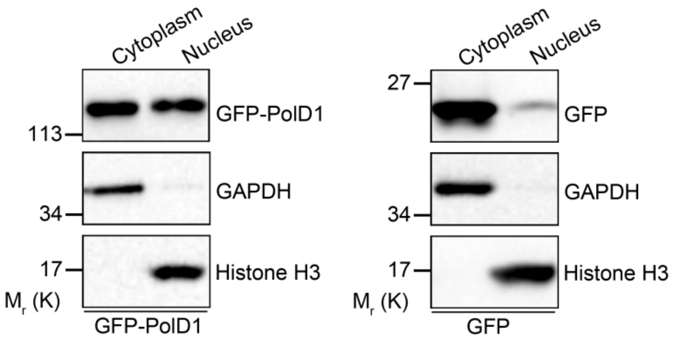

Supplementary Figure 5 Subcellular distribution of GFP-PolD1 and GFP. RPE1 cells transfected with GFP-PolD1 or GFP were lysed and the lysates were fractionated into cytoplasmic (Cytoplasm) and nuclear (Nucleus) fractions. An equal proportion of each fraction was immunoblotted with anti-GFP, anti-GAPDH, and anti-Histone H3.

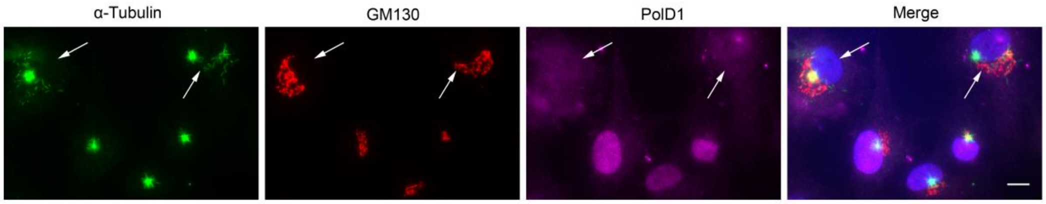

Supplementary Figure 6 Golgi size and Golgi-associated microtubule nucleation are inversely correlated with the PolD1 level in RPE1 cells. After microtubule depolymerization at 0°C, cells were shifted to 37°C to allow microtubule regrowth for 1 min, after which the cells were immunostained for microtubules (anti-α-tubulin), GM130, and PolD1. Hoechst 33258 was used to stain DNA. Arrows indicate cells containing low levels of PolD1. The images shown are representative of at least 3 experiments. Scale bar, 10 μm.

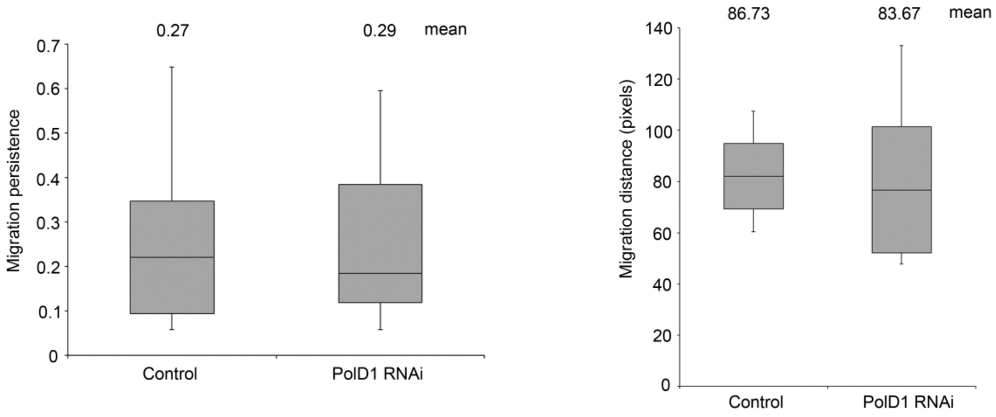

Supplementary Figure 7 PolD1 knockdown does not affect random migration of cells. The persistence and the distance of random migration of control and PolD1 siRNA-transfected cells were measured and are shown here as box-and-whisker plots (n = 50 cells per condition). Boxes represent the 25th and 75th percentiles and whiskers represent the 10th and 90th percentiles of persistence or distance.

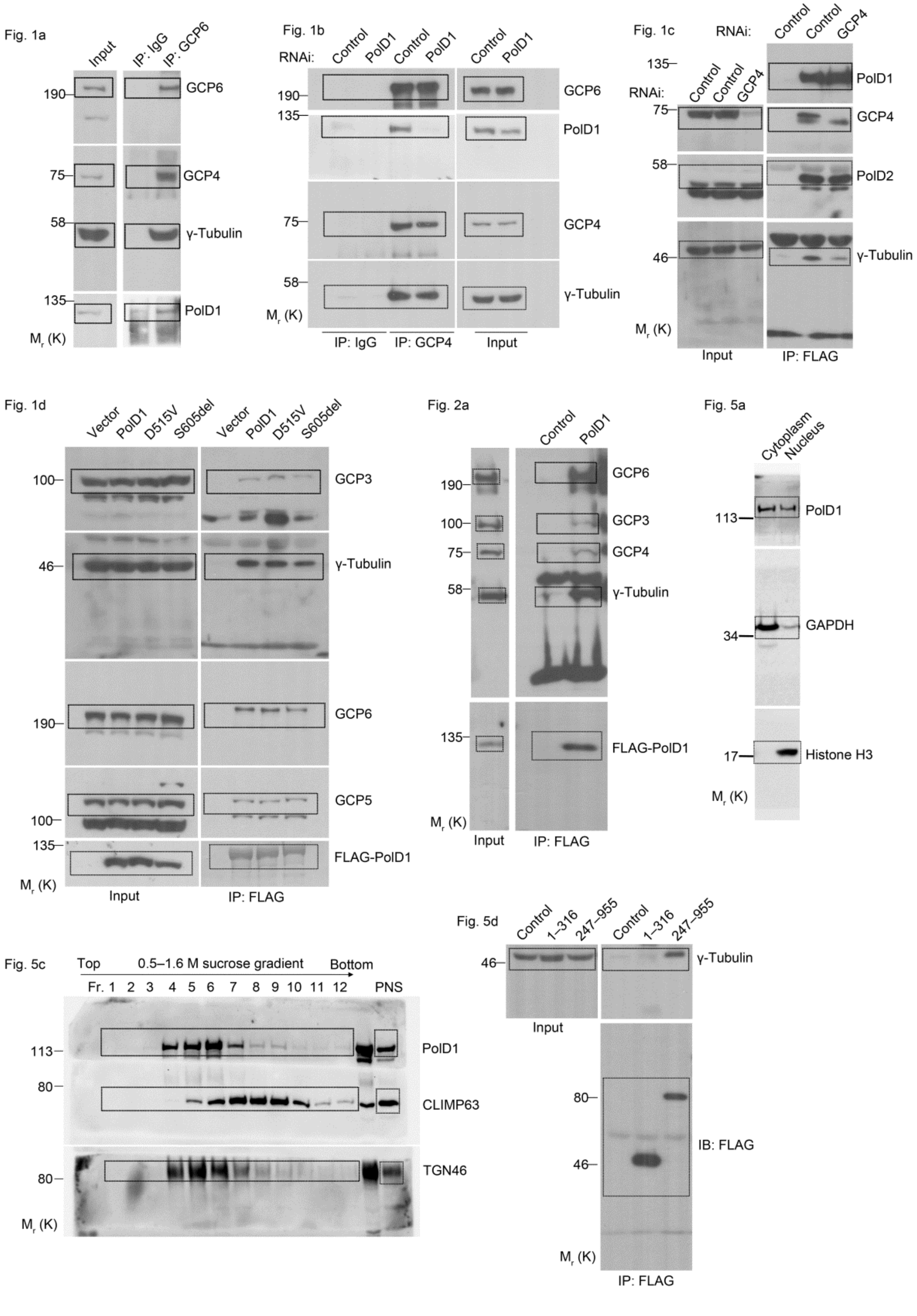

Supplementary Figure 8 Uncropped Western Blot images.

PolD1 peptides identified by mass spectrometry

No.	Expectation value	Peptide
1	1.2×10^{-2}	AEAVLLR
2	8.5×10^{-3}	VVSQLLR
3	9.3×10^{-3}	LLEQGIR
4	3.9×10^{-4}	VGGLLAFAK
5	7.0×10^{-3}	ELNLAISR
6	3.6×10^{-2}	TPTGDEFVK
7	3.9×10^{-3}	QAHVELAER
8	3.3×10^{-4}	IDISQLVITK
9	4.3×10^{-3}	IFEPILGEGR
10	7.4×10^{-4}	LGLTEDQFIR
11	9.9×10^{-4}	DLEDQEQLLR
12	5.5×10^{-3}	VTGVPLSYLLSR
13	5.5×10^{-4}	LMVLVNAVEMAR
14	1.4×10^{-3}	VQMDMLQVLLR
15	8.4×10^{-7}	FGVSSVAEAMALGR
16	3.3×10^{-2}	QAMHEGLLMPVVK
17	1.2×10^{-3}	VSANAVYGFTGAQVGK
18	1.4×10^{-4}	EAADWVSGHFPSPIR
19	1.0×10^{-6}	SEGGEDYTGATVIEPLK
20	9.5×10^{-6}	SYTLNAVSFHFLGEQK
21	2.6×10^{-2}	VQTFPFLGR
22	3.7×10^{-3}	VYFPYLLISK
23	4.4×10^{-2}	EVSHLNALEER
24	2.6×10^{-6}	DLEDQEQLLR
25	8.2×10^{-5}	VSANSVYGFTGAQVGK
26	3.1×10^{-2}	EDVQHSIITDLQNGNDQTR

Supplementary Table 1 PolD1 peptides identified using mass spectrometry. The expected values were calculated by using MASCOT software with a cut-off value of 5.0×10^{-2}. The sequence coverage of PolD1 was 26.8%. The results shown are representative of 3 isolation experiments.

Source data for Figure 2b

Microtubule numbers (%)	0 nM PolD1	56 nM PolD1	111 nM PolD1	222 nM PolD1	267 nM PolD1	356 nM PolD1
Average	100	79.72	41.94	16.13	13.96	9.6
s.d.	0	16.41	11.17	1.64	1.93	2.31
s.e.m.	0	5.19	3.53	0.52	0.61	0.73

Supplementary Table 2 Source data for Figure 2b.

Source data for Figure 2c

Microtubule numbers (%)	Buffer	γTuRC	γTuRC+ PolD1	γTuRC+ D515V	γTuRC+ S605del	γTuRC+ PolD2
Average	0.76	100	15.3	6.27	3.47	98.18
s.d.	0.52	0	7.21	2.26	1.99	1.08

Supplementary Table 3 Source data for Figure 2c.

Source data for Figure 3c

Microtubule numbers normalized by Golgi mass	GFP	PolD1	D515V	S605del
Exp#1	3.8	0.92	0.85	0.74
Exp#2	4.66	0.64	0.76	0.89
Exp#3	4.83	0.59	0.58	0.64
Average	4.43	0.72	0.73	0.76
s.d.	0.55	0.18	0.14	0.13
P-value		0.00038	0.00036	0.00036

Supplementary Table 4 Source data for Figure 3c.

Source data for Figure 4

Microtubule numbers normalized by Golgi mass	Control	PolD1 RNAi
Exp#1	2.94	18.22
Exp#2	2.18	15.56
Exp#3	4.60	21.32
Average	3.24	18.37
s.d.	1.24	2.89
P-value		0.00089

Supplementary Table 5 Source data for Figure 4.

Source data for Figure 5e

Microtubule numbers normalized by Golgi mass	GFP	247–955
Exp#1	5.50	0.35
Exp#2	3.50	0.54
Exp#3	4.67	0.33
Average	4.56	0.41
s.d.	1.00	0.15
P-value		0.0021

Supplementary Table 6 Source data for Figure 5e.

Source data for Figure 6a (Golgi area)

Golgi area (%)	Control	PolD1 RNAi
Exp#1	100	159.09
Exp#2	100	132.28
Exp#3	100	168.20
Average	100	152.48
s.d.	0	18.67
P-value		0.0387

Supplementary Table 7 Source data for Figure 6a (Golgi area).

Source data for Figure 6a (Golgi compactness)

Golgi compactness (%)	Control	PolD1 RNAi
Exp#1	70.95	52.86
Exp#2	68.5	54.22
Exp#3	70.91	57.47
Average	70.12	54.85
s.d.	1.4	2.37
P-value		0.0017

Supplementary Table 8 Source data for Figure 6a (Golgi compactness).

Source data for Figure 6a (Microtubule intensity)

Microtubule intensity (%)	Control	PolD1 RNAi
Exp#1	100	166.47
Exp#2	100	155.35
Exp#3	100	147.92
Average	100	156.58
s.d.	0	9.33
P-value		0.0089

Supplementary Table 9 Source data for Figure 6a (Microtubule intensity).

Source data for Figure 6c (Average size of dispersed Golgi particles)

Control					
Average size of dispersed Golgi particles (%)	0 min	7 min	15 min	22 min	30 min
Exp#1	100	143.87	183.87	176.85	129.63
Exp#2	100	132.28	186.97	161.69	135.35
Exp#3	100	122.43	170.00	139.37	161.73
Average	100	132.86	180.28	159.31	142.24
s.e.m.	0	6.20	5.22	10.89	9.89
PolD1 RNAi					
Average size of dispersed Golgi particles (%)	0 min	7 min	15 min	22 min	30 min
Exp#1	100	126.98	160.85	162.14	151.58
Exp#2	100	135.99	192.48	167.29	156.01
Exp#3	100	130.71	155.59	148.76	145.13
Average	100	131.23	169.64	159.40	150.91
s.e.m.	0	2.62	11.52	5.52	3.16

Supplementary Table 10 Source data for Figure 6c (Average size of dispersed Golgi particles).

Source data for Figure 6c (Total area of dispersed Golgi particles)

Control				
Total area of dispersed Golgi particles (%)	0 min	15 min	30 min	60 min
Exp#1	100	52.83	19.25	2.14
Exp#2	100	44.27	16.90	6.22
Exp#3	100	56.31	9.16	5.05
Average	100	51.14	15.10	4.47
s.e.m.	0	3.58	3.05	1.21
PolD1 RNAi				
Total area of dispersed Golgi particles (%)	0 min	15 min	30 min	60 min
Exp#1	100	53.74	32.18	23.64
Exp#2	100	76.02	40.66	26.50
Exp#3	100	57.49	44.65	25.89
Average	100	62.42	39.16	25.34
s.e.m.	0	6.88	3.68	0.087

Supplementary Table 11 Source data for Figure 6c (Total area of dispersed Golgi particles).

Source data for Figure 7a

Reoriented Golgi (% cells)	0 h	3 h	5 h	8 h
Control	24.07	46.24	70.06	84.21
s.e.m.	1.05	1.59	1.67	1.76
PolD1 RNAi	25.37	31.28	44.64	52.49
s.e.m.	3.20	1.09	1.43	2.38

Supplementary Table 12 Source data for Figure 7a.

Source data for Figure 7c

Scratch area (%)						
Control	Exp#1	Exp#2	Exp#3	Average	s.d.	s.e.m.
0 min	100	100	100	100	0	0
50 min	97.81	96.86	97.13	97.27	0.49	0.28
100 min	95.86	95.29	94.50	95.22	0.69	0.40
150 min	94.40	91.48	92.34	92.74	1.5	0.87
200 min	92.21	89.69	88.28	90.06	1.99	1.15
250 min	89.78	87.89	83.49	87.06	3.23	1.86
300 min	86.13	85.65	82.30	84.69	2.09	1.21
350 min	81.51	81.84	76.56	79.97	2.96	1.71
400 min	76.40	76.46	72.73	75.19	2.14	1.23
450 min	73.24	73.32	70.10	72.22	1.84	1.06
500 min	67.64	66.59	61.96	65.40	3.02	1.74
550 min	65.69	58.52	57.90	60.70	4.33	2.50
600 min	55.72	55.61	53.59	54.97	1.20	0.69
650 min	51.58	47.76	48.33	49.22	2.06	1.19
700 min	47.20	43.95	41.39	44.18	2.91	1.68
750 min	41.36	40.13	39.47	40.32	0.96	0.55
800 min	36.25	35.65	34.69	35.53	0.79	0.46
850 min	35.28	30.04	28.23	31.18	3.66	2.11
900 min	28.71	24.44	23.68	25.61	2.71	1.56
950 min	25.79	19.28	18.18	21.09	4.11	2.37
1000 min	24.09	13.45	11.00	16.18	6.96	4.02
1050 min	19.71	8.52	6.22	11.48	7.22	4.17
1100 min	7.54	4.93	0	4.16	3.83	2.21
1150 min	0	0	0	0	0	0
1185 min	0	0	0	0	0	0
Scratch area (%)						
PolD1 RNAi	Exp#1	Exp#2	Exp#3	Average	s.d.	s.e.m.
0 min	100	100	100	100	0	0
50 min	98.25	97.74	97.67	97.89	0.32	0.18
100 min	93.77	96.61	95.60	95.33	1.44	0.83
150 min	91.27	94.36	93.26	92.96	1.56	0.90
200 min	88.78	92.78	90.93	90.83	2.00	1.16
250 min	85.04	90.29	89.64	88.32	2.86	1.65
300 min	83.04	85.78	89.12	85.98	3.04	1.76
350 min	82.54	81.72	87.56	83.94	3.17	1.83
400 min	77.06	76.52	83.42	79.00	3.84	2.22
450 min	73.32	72.69	80.57	75.52	4.38	2.53
500 min	67.08	71.11	77.20	71.80	5.10	2.94
550 min	64.09	65.91	75.65	68.55	6.21	3.59
600 min	61.60	64.79	73.58	66.65	6.20	3.58
650 min	58.10	61.63	70.21	63.31	6.23	3.59
700 min	54.61	59.59	64.25	59.49	4.82	2.78
750 min	50.62	57.79	62.69	57.04	6.07	3.50
800 min	46.13	55.08	60.10	53.77	7.08	4.09
850 min	44.64	51.69	54.66	50.33	5.15	2.97
900 min	40.65	48.76	44.56	44.66	4.06	2.34
950 min	37.66	46.50	41.19	41.78	4.45	2.57
1000 min	34.16	41.53	33.42	36.37	4.49	2.59
1050 min	30.17	36.57	27.72	31.49	4.57	2.64
1100 min	24.94	33.41	23.06	27.13	5.51	3.18
1150 min	22.44	28.22	21.24	23.97	3.73	2.15
1185 min	20.45	25.96	19.95	22.12	3.34	1.93

Supplementary Table 13 Source data for Figure 7c.

Source data for Supplementary Figure 3b

Fluorescence intensity	CDK5RAP2	γ-Tubulin		CDK5RAP2	γ-Tubulin
Control	15397.92	5641.19	PolD1 RNAi	29865.60	6455.75
	25500.05	6386.57		18146.35	5284.75
	24528.86	4851.38		15140.48	3685.75
	9135.61	3830.20		15619.12	4293.30
	19956.90	3708.27		23627.30	4063.15
	15844.01	5382.11		23987.22	5090.35
	11127.27	2926.14		14162.32	3986.69
	8079.59	2522.30		9399.89	1996.79
	14274.86	3573.96		12331.27	2834.87
	15805.22	4301.15		26555.61	6692.04
	10713.21	4023.67		17373.28	5888.37
	12659.07	5171.24		18301.46	5160.69
	17027.33	5446.39		23774.10	6581.43
	18457.69	5898.58		14982.12	4862.45
	13180.98	3866.29		13932.20	4220.90
	13763.31	3532.35		6918.91	1987.48
	12174.30	3704.60		10468.91	3294.48
	12101.72	3098.28		12476.66	3742.81
	18617.68	5244.38		16218.30	4759.63
	9118.88	2814.68		12063.02	4185.21
	10542.85	3414.47		14213.36	5418.44
	16327.93	4468.26		11388.36	3640.30
	27386.17	5360.82		8710.80	3205.89
	15931.41	4251.44		14320.59	3583.20
	21364.58	5869.31		12849.97	3764.40
	19014.89	4233.27		13712.85	4325.86
	19963.06	4170.08		17778.24	3116.56
	14366.29	2871.46		16281.22	3875.18
	21286.50	4791.56		22400.05	6048.56
	24130.88	4802.57		23938.44	3709.45
	17617.95	4520.73		21639.05	5839.43
	19835.59	5555.35		20221.21	4060.36
	21707.64	6764.36		16737.82	5551.06
	15464.57	4626.75		16383.78	4004.83
	18963.48	4847.63		16105.51	4807.74
	18923.50	4169.62		15926.83	5102.44
	9680.16	2153.91		16128.97	4371.84
	12549.61	3330.04		12314.19	3559.97
	13807.17	3889.36		21305.59	4046.55
	7479.62	3172.33		23551.29	3643.04
	21575.90	4523.88		19275.98	3456.95
	12167.58	3114.62		9861.74	2770.92
	24907.41	5137.36		20674.39	4775.78
	26164.81	6121.70		10441.27	3715.06
	26750.50	5291.43		20524.36	3271.28
	16576.15	4945.06		15339.13	4497.88
	25509.84	5117.88		23457.76	5577.35
	19387.79	2735.78		14417.82	3627.01
	16349.12	3820.26		20112.38	5083.56
	16719.98	4010.03		15645.10	2970.47
Average	16998.35	4360.10		16820.04	4289.77
s.d.	5273.19	1072.32		4991.78	1102.03
P-value				0.8625	0.7470

Supplementary Table 14 Source data for Supplementary Figure 3b.

Source data for Supplementary Figure 3c

Microtubule numbers normalized by Golgi mass	PolD1 RNAi	PolD1&AKAP450 RNAi
Exp#1	15.75	0.50
Exp#2	16.83	0.89
Exp#3	16.25	0.88
Average	16.28	0.75
s.d.	0.54	0.22
P-value		1.34×10^{-6}

Supplementary Table 15 Source data for Supplementary Figure 3c.

Source data for Supplementary Figure 7

Migration persistence	Control	PolD1 RNAi
90%	0.65	0.60
75%	0.35	0.39
Median	0.22	0.18
25%	0.09	0.12
10%	0.06	0.06
Mean	0.27	0.29
Migration distance (pixels)	Control	PolD1 RNAi
90%	109.05	137.41
75%	96.51	105.70
Median	82.14	76.74
25%	69.33	52.16
10%	60.39	47.70
Mean	86.73	83.67

Supplementary Table 16 Source data for Supplementary Figure 7.
